# Density artefacts at interfaces caused by multiple time-step effects in molecular dynamics simulations

**DOI:** 10.12688/f1000research.16715.3

**Published:** 2019-03-08

**Authors:** Dominik Sidler, Marc Lehner, Simon Frasch, Michael Cristófol-Clough, Sereina Riniker

**Affiliations:** 1 Laboratory of Physical Chemistry, ETH Zürich, Zurich, 8093, Switzerland

**Keywords:** Multiple Time-Step Integration, Molecular Dynamics, Resonance Artefacts, RESPA, Twin-range, Liquid Phase Interfaces, Thermostat

## Abstract

**Background: **Molecular dynamics (MD) simulations have become an important tool to provide insight into molecular processes involving biomolecules such as proteins, DNA, carbohydrates and membranes. As these processes cover a wide range of time scales, multiple time-step integration methods are often employed to increase the speed of MD simulations. For example, in the twin-range (TR) scheme, the nonbonded forces within the long-range cutoff are split into a short-range contribution updated every time step (inner time step) and a less frequently updated mid-range contribution (outer time step). The presence of different time steps can, however, cause numerical artefacts.

**Methods: **The effects of multiple time-step algorithms at interfaces between polar and apolar media are investigated with MD simulations. Such interfaces occur with biological membranes or proteins in solution.

**Results: **In this work, it is shown that the TR splitting of the nonbonded forces leads to artificial density increases at interfaces for weak coupling and Nosé-Hoover (chain) thermostats. It is further shown that integration with an impulse-wise reversible reference system propagation algorithm (RESPA) only shifts the occurrence of density artefacts towards larger outer time steps. Using a single-range (SR) treatment of the nonbonded interactions or a stochastic dynamics thermostat, on the other hand, resolves the density issue for pairlist-update periods of up to 40 fs.

**Conclusion: **TR schemes are not advisable to use in combination with weak coupling or Nosé-Hoover (chain) thermostats due to the occurrence of significant numerical artifacts at interfaces.

## Introduction

**Figure 1.**
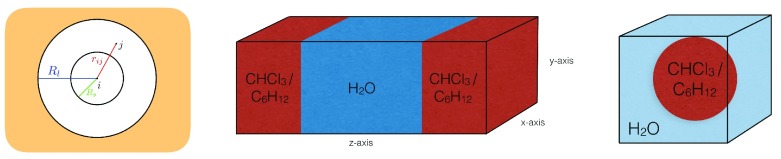
Simulated Systems. Left: Schematic illustration of the *twin-range* (TR) setup with the inner cutoff *R _s_* and the outer cutoff *R _l_*. Interactions beyond *R _l_* are not calculated explicitly. Middle: Schematic illustration of the planar layer systems. Right: Schematic illustration of the systems with a droplet in a water box.

To describe the dynamic processes of biomolecules, such as proteins, DNA, carbohydrates and membranes, molecular dynamics (MD) simulations have been proven to be a powerful technique to provide insights at the atomic level. In MD simulations, physical processes that involve a wide range of time scales have to be considered appropriately. However, the accurate and efficient treatment of various time scales presents a notoriously difficult problem due to resonance artefacts arising from separation into distinct numerical subproblems ^1, 2^. Over the past decades, various methods have been proposed to mediate the issue and to speed up the simulation substantially. For example, fast vibrational modes (e.g. covalent bonds) can be removed completely by constraining the motion, e.g. using SHAKE ^3^, SETTLE ^4^, M-SHAKE ^5^, LINCS ^6^, or FLEXSHAKE ^7^. Alternatively, multiple time-step (MTS) integration is an efficient approach to accommodate motions on different time scales. In the *twin-range* (TR) scheme ^8– 10^, the pairwise nonbonded forces ${\text{f}}_{i}^{\mathrm{nb}}$ acting on particle *i* are split into a short-range contribution from particles *j* within the cutoff *R _s_* , and a mid-range contribution from particles *j* between *R _s_* and the long-range cutoff *R _l_* (see Figure 1),

$${\text{f}}_{i}^{\mathrm{nb}}(t)={\text{f}}_{i}^{\mathrm{nb},s}(t)+{\text{f}}_{i}^{\mathrm{nb},m}(t).\;(1)$$

with

$${\text{f}}_{i}^{\mathrm{nb},s}(t)=\sum_{j\in N(r_{ij}\leq R_{s})}{\text{f}}_{ij}^{\mathrm{ele}}+{\text{f}}_{ij}^{\mathrm{vdw}},\;(2)$$

$${\text{f}}_{i}^{\mathrm{nb},m}(t)=\sum_{j\in N(R_{s}<r_{ij}\leq R_{l})}{\text{f}}_{ij}^{\mathrm{ele}}+{\text{f}}_{ij}^{\mathrm{vdw}}\cdot \;(3)$$

The short-range forces ${\text{f}}_{i}^{\mathrm{nb},s}$ are updated every time step ∆ *t* ( *inner* time step), while the mid-range forces ${\text{f}}_{i}^{\mathrm{nb},m}$ are updated every *n _m_*-th time step, *n _m_*∆ *t* with *n _m_* ≥ 1 ( *outer* time step). Long-range electrostatic interactions beyond the cutoff radius *R _l_* can be included using the (an)isotropic reaction-field methods (RF or ARF) ^11, 12^, or lattice-sum methods ^13, 14^. Although theoretically possible, the TR scheme is generally not used together with lattice-sum methods. Simulations with (A)RF typically use larger cutoffs *R _l_* than simulations with PME (e.g. 1.4 nm for the GROMOS force field ^15^ compared to 0.9 nm for the AMBER force field ^16^), which in the past encouraged the use of a TR scheme to reduce the computational cost.

In practice, for each particle *i* two pairlists are generated, which track the neighbouring short-range and mid-range particles *j*. To be consistent, the period of updating both pairlists *n _p_∆t* should be equal to the period of updating the mid-range forces *n _m_*∆ *t*. In the following, the TR scheme is defined by *R _s_ < R _l_* and *n _m_* = *n _p_* > 1, and the *single-range* (SR) scheme by *R _s_* = *R _l_* , *n _m_* = 1 and *n _p_* ≥ 1, i.e. there is only one pairlist for SR.

Splitting the forces into different contributions leaves some freedom of how to consider them in the integration. There are two main strategies for the inclusion of the outer contributions: (i) constant mid-range forces application (CFA) ${\text{f}}_{i}^{\mathrm{nb},m(\mathrm{CFA})}(t)$ at every inner time step ∆ *t*, or (ii) using a reversible reference system propagator algorithm (RESPA) ^8– 10^, which is based on the Trotter factorisation of the Liouville propagator ^17^. In the case of RESPA, scaled mid-range forces ${\text{f}}_{i}^{\mathrm{nb},m(\mathrm{RESPA})}(t)$ are typically applied impulse-wise, i.e. only at every outer time step *n _m_*∆ *t*. Note that in this work the leap-frog scheme ^18^ is used for the integration of the equations of motion, which is similar ^19^ to velocity-Verlet ^20^ as typically employed with RESPA. The resulting discrete integration scheme can be written as follows,

$$\begin{array}{l} {\text{x}}_{i}(t+\Delta t)={\text{x}}_{i}(t)+{\text{v}}_{i}\left(t+\frac{1}{2}\Delta t\right)\;\Delta t,\\ {\text{v}}_{i}\left(t+\frac{1}{2}\Delta t\right)={\text{v}}_{i}\left(t-\frac{1}{2}\Delta t\right)+\frac{1}{m_{i}}\left({\text{f}}_{i}^{\text{nb},\text{s}}(t)+{\text{f}}_{i}^{\text{nb,m(}\cdot \text{)}}(t)\right)\Delta t,\\ \;{\text{f}}_{i}^{\text{nb,m(CFA)}}(t):\;={\text{f}}_{i}^{\text{nb,m}}(t-t\;\mathrm{mod}\;n_{m}\Delta t),\\ \;{\text{f}}_{i}^{\text{nb,m(RESPA)}}(t):\;=\left\{ \begin{array}{l} n_{m}{\text{f}}_{i}^{\text{nb,m}}(t),\;t\;\mathrm{mod}n_{m}\Delta t=0\\ \;0,\;\text{else}. \end{array}\right. \end{array}$$

Note that for simplicity, the notation is restricted to the microcanonical case and it is implicitly assumed that *t* mod ∆ *t*: = ∆ *t*(n mod 1) = 0, i.e. the time *t* = *n*∆ *t* is discretized with respect to ∆ *t*, where mod denotes the modulo operation.

Although computationally efficient, MTS algorithms have the disadvantage of introducing numerical artefacts. Due to nonlinearity, it is notoriously difficult to formulate general physical or chemical conditions, which favour or suppress the build-up of these undesired resonances. Instead, specialised thermostats have been proposed as a remedy, which suppress resonance artefacts from MTS integration, for example by imposing isokinetic constraints on every degree of freedom ^21– 23^, or by selectively targeting the high-frequency modes using a colored noise thermostat ^24^. In practice, however, standard thermostats like weak-coupling ^25^, Nosé-Hoover (chain) ^26– 28^, or stochastic thermostats ^29– 31^ remain widely used in combination with a moderate integration time-step difference between fast and slow motions, as this should limit resonance artefacts to an acceptable value.

In this work, artefacts due to MTS integration are investigated with the main focus on a water-chloroform layer system using different thermostats ( Figure 1). This system represents a simplified version of a biological membrane, for which an accurate simulation is of high interest in biology. The impact of splitting the nonbonded interactions into a short-range and mid-range contribution on the density profile normal to the interface and the kinetic energy distribution is investigated. In addition, a planar system consisting of cyclohexane and water as well as corresponding droplet systems are investigated. These additional setups allow to assess the impact of different solvents as well as different interface geometries on the formation of MTS artefacts. Overall, the artefacts are larger than those observed for more well-behaved, homogeneous systems ^32, 33^.

## Methods

For all simulations, the GROMOS software package ^34^ version 1.4.0 was used with the GROMOS 53A6 force field ^15^. Periodic boundary conditions were applied. Newton’s equations of motion were integrated using the leap-frog scheme ^18^ with an inner time step of 2 fs. Different outer time steps for the pairlist update or mid-range force evaluation were used (i.e. *n _m_* , *n _p_* ∈ [1, 20]). If not indicated otherwise, the SR scheme corresponds to a midrange force evaluation every 2 fs ( *n _m_* = 1) and a pairlist update every 10 fs ( *n _p_* = 5). The TR scheme typically corresponds to a mid-range force evaluation in combination with a pairlist update every 10 fs ( *n _m_* = *n _p_* = 5). If not stated otherwise, the CFA method was used for the mid-range force contributions. The inner cutoff radius *R _s_* was set to 0.8 nm and the outer cutoff radius *R _l_* to 1.4 nm. The center of mass motion was stopped every 1 ps. The coordinates were saved every 0.2 ps and the energies every 0.1 ps. Bond lengths were constrained using the SHAKE algorithm with a geometric tolerance of 10 ^−4^. Four different thermostats were tested, i.e. weak coupling ^25^ (WC), Nosé-Hoover ^26, 27^ (NH), Nosé-Hoover chain ^28^ (NHchain), and stochastic dynamics (SD) i.e. a Langevin thermostat ^35^. Different temperature baths were used for different solvents. The coupling time *τ* was set to 0.1 ps for all non-stochastic thermostatting methods. A chain length of three was employed for the NHchain thermostat. For SD, a friction coefficient γ = 10 ps ^-1 ^was used. A standard homogeneous RF approach ^11^ with a charge-group based pairlist algorithm was used (each solvent molecule is a single charge group) to mimic long-range electrostatic interactions beyond the cutoff *R _l_*. The dielectric permittivity was set to 78.5, which corresponds to the experimentally measured value of water ^36^.

### Systems

The MTS investigation was performed mainly using a water-chloroform layer, in combination with three auxiliary systems consisting of a water-cyclohexane layer, a chloroform sphere in a water box, and a cyclohexane sphere in a water box. An illustration of the setups is shown in Figure 1. With these additional systems, different geometries of the interface as well as different chemical compositions were tested. An overview of the simulated systems can be found in Table 1. Corresponding snapshots are provided in Figure S1 ( Supplementary File 1).

**Table 1.** Overview of the system dimensions and simulation times.

Setup	Phase 1 [#molecules]	Phase 2 [#molecules]	Box (x, y, z) [nm]	Time [ns]
Layer	2002 CHCl _3_	8129 H _2_O	(4.1, 4.1, 30.2)	4
Layer	6750 C _6_H _12_	39’366 H _2_O	(8.5, 8.5, 33.9)	1
Droplet	847 CHCl _3_	59’484 H _2_O	(12.2, 12.2, 12.2)	1
Droplet	619 C _6_H _12_	59’018 H _2_O	(12.2, 12.2, 12.2)	1

The main system consisted of 2002 chloroform (CHCl _3_) ^37^ molecules and 8129 SPC ^38^ water molecules within an elongated rectangular box of 30.2 nm length in *z*-direction. The initial coordinates were generated by combining pre-equilibrated (under NPT conditions) boxes of chloroform and water. The combined system was equilibrated for 1 ns using the SR scheme under NVT conditions. Although this meant a slightly too small box size for the NVT conditions, we chose this procedure to avoid effects from the MTS scheme and/or the barostatting method. The simulation length of the production simulations was 1–4 ns, performed under NVT conditions. For the water-cyclohexane layer system, 6750 cyclohexane (C _6_H _12_) and 39’366 water molecules were used in an elongated rectangular box of 33.85 nm length in *z*-direction. Coordinates were generated by combining pre-equilibrated boxes of cyclohexane and water. This system was simulated under NVT as well as NPT conditions (using a Berendsen barostat ^25^ with a coupling time *τ _b_* = 0.5 ps). The same starting coordinates were used for all production runs to allow direct comparison, followed by 200 ps of equilibration under the given conditions.

The droplet systems consisted of a droplet of about 3 nm radius, solvated in a box of 12.2 nm length. This setup led to a droplet with 847 CHCl _3_ molecules in 59’484 SPC water molecules, or a droplet with 619 C _6_H _12_ molecules solvated in 59’018 SPC water molecules, respectively. As the convergence towards the numerical artefacts was found to be very fast, all auxiliary systems were simulated without initial equilibration for 1 ns.

### Analysis

For the spatially resolved density analysis, a program was developed using the data structures provided by the GROMOS++ collection of programs ^39^. For the planar systems, the densities were obtained from 100 equidistant bins along the z-axis, e.g. for the main chloroform-water system the averaging bin volume was 5.0 nm ^3^. The energy distributions were calculated by post-processing the output of the GROMOS++ program ene_ana accordingly. For the density calculations of the spherical interfaces, a slightly more sophisticated analysis approach was necessary. In order to obtain a radial density profile, the molecules were assigned to equidistant concentric spherical shells with respect to the center of geometry of the droplet. However, for the droplet it was difficult to define consistently a distance to the surface, because the interface may deviate substantially from its ideal spherical shape during simulation. In our analysis, the center of mass was chosen as an approximation of the center of geometry, which was then shifted (if necessary) in order to account for periodic boundary conditions, i.e. to ensure that the droplet remained at the center of the periodic box. Note that the volume of the spherical shells scales quadratically with the radius, which increases the uncertainty of the estimator substantially close to the center of the droplet.

## Results & discussion

**Figure 2.**
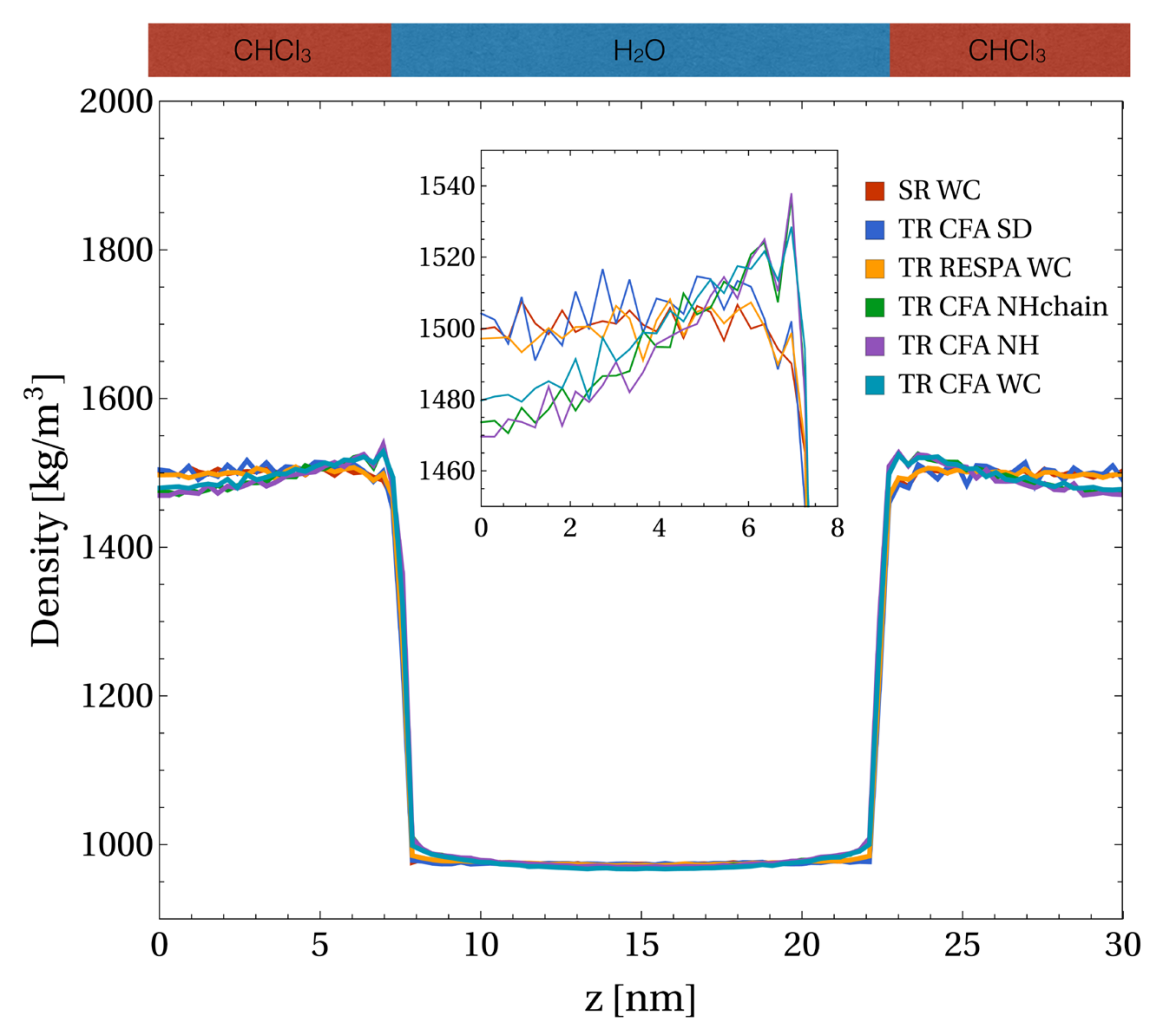
Profile of the density *ρ* in the water-chloroform layer system with respect to the layer normal *z*. For the five setups with the twin-range (TR) scheme, the mid-range forces were updated with the same frequency as the pairlist (i.e. *n _m_* = *n _p_* = 5). For the single-range (SR) scheme, the mid-range forces were evaluated every time step ∆ *t* (i.e. *n _m_* = 1), whereas the pairlist was updated every 5th step (i.e. *n _p_* = 5). A weak-coupling (WC), Nosé-Hoover (NH), Nosé-Hoover chain (NHchain), or a stochastic (SD) thermostat were used.

**Figure 3.**
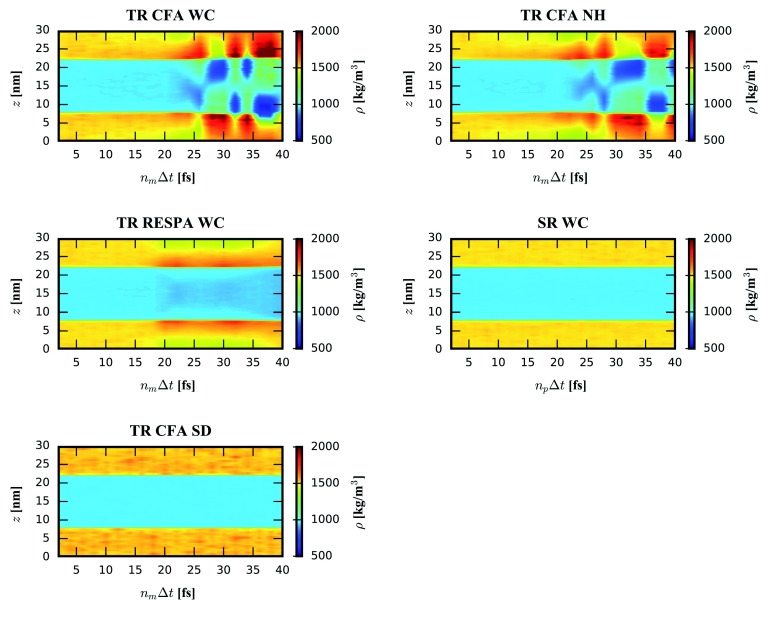
Density profile with respect to the layer normal *z* and the pairlist update period *n _p_∆t*. For the single-range (SR) scheme, the mid-range forces were evaluated every time step ∆ *t* (i.e. *n _m_* = 1). For the four setups with the twin-range (TR) scheme, the mid-range forces were updated with the same period as the pairlist (i.e. *n _p_* = *n _m_*). The TR scheme was applied in a constant (CFA) or impulse-wise (RESPA) manner. A weak-coupling (WC), Nosé-Hoover (NH), or a stochastic dynamics (SD) thermostat were used. Note that the density range is chosen to visualize the major artefacts occurring for *n _m_*∆ *t* > 15 fs. A visualisation of the minor artefacts around *n _m_*∆ *t ≈* 10 fs can be seen in Figure 4.

The density profile of the water-chloroform system was determined along the *z*-axis normal to the layer. As can be seen in Figure 2, the splitting of the forces into a short-range and a less frequently updated mid-range contribution (given in Equation (1)) using the TR CFA scheme with *n _m_* = *n _p_* = 5 led to a significant density increase in chloroform close to the interface for WC, NH and NHchain thermostats. A corresponding graph with the separate densities of the two phases is given in Figure S2, Supplementary File 1. The small density fluctuations in Figure 2 are due to the relative orientation of the molecules at the interface in combination with the granularity of the molecules and the binning. On the contrary, no significant density increase was found for SD for pairlist-update periods up to 40 fs as shown in Figure 3. However, SD appears to cause slightly more pronounced local density fluctuations in the chloroform phase compared to the non-stochastic thermostats for the chosen simulation time of 1 ns, independent of the pairlist-update period.

**Figure 4.**
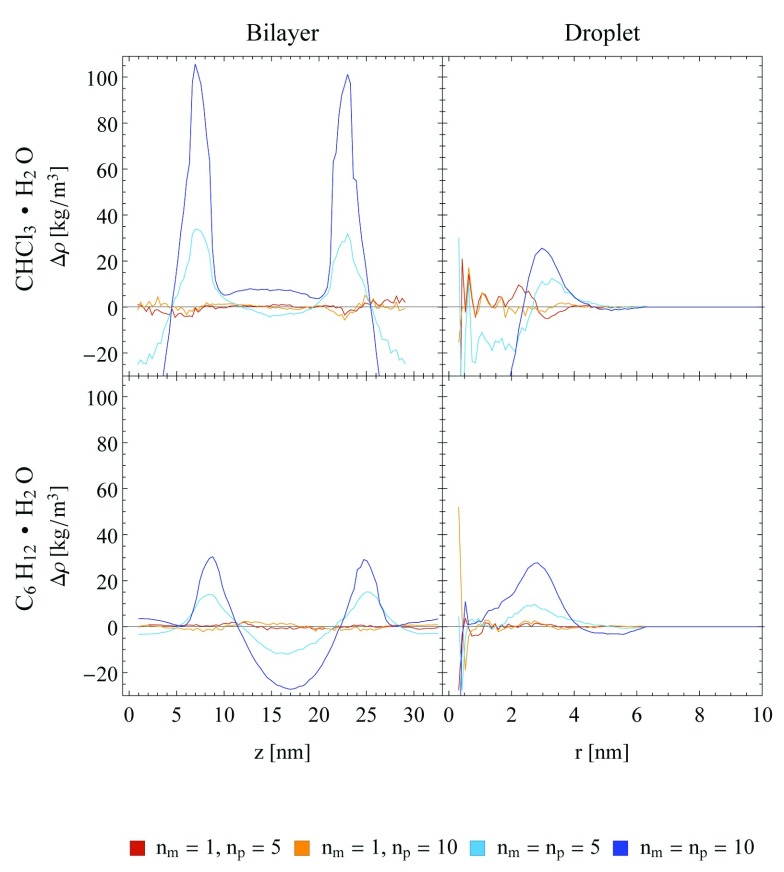
Spacial density difference profiles with respect to a SR reference simulation ( *n _m_* = *n _p_* = 1) under NVT conditions for layer systems (left) and droplet systems (right) with water-chloroform composition (top) or water-cyclohexane composition (bottom). The Nosé-Hoover (NH) thermostat was used in all setups. The single-range (SR) scheme (red and orange lines) and the twin-range scheme with constant-force application (TR CFA, light and dark blue lines) and two different update frequencies ( *n _p_* = 5, 10) are compared. Running averages with a window of seven data points are shown.

For the non-stochastic thermostats, the density increase of the TR setup was accompanied by a density decrease further away from the interface due to the constant volume conditions. An analysis of the density resonance pattern with respect to the outer time step *n _m_*∆ *t* is shown in Figure 3. Note that the density artefacts are not perfectly symmetric with respect to the layer normal due to NVT conditions and finite layer thickness. The asymmetry for large outer time steps is due to the small thickness of the layers, which enables aggregation at one interface and depletion at the other interface. It can be seen that the TR CFA scheme leads to large resonance artefacts beyond *n _m_*∆ *t ≈* 20 fs. On the other hand, evaluating the mid-range forces every step while keeping the pairlist update every fifth step (i.e. SR scheme with *n _m_* = 1 and *n _p_* = 5) resolved the issue almost entirely for all investigated thermostats ( Figure 2 and Figure 3, data not shown for NHchain). The minor differences between SR schemes with different update frequencies can only be seen in density difference plots ( Figure 4) with respect to the SR reference run ( *n _m_* = *n _p_* = 1).

These findings indicate that the observed density increase among non-stochastic thermostats is mainly caused by resonance artefacts due to the CFA MTS integration. This argumentation is supported by the pairlist error analysis shown in Figure S3, Supplementary File 1. The change in the pairlist after 10 fs is only on the order of 1%, and, even more importantly, it scales sublinearly with *n _m_*. Thus, the error from the pairlist cannot explain the substantial density increases seen in Figure 3. Instead, resonance effects lead to the build-up of the observed density artefacts over time, until a local equilibrium configuration is reached at the interface. This was found to be accompanied by a local change in the diffusion of the molecules at the interface. The mean residence time with respect to the layer normal *z* shows that regions of high density imply a locally reduced diffusivity of the molecules and *vice versa* ( Figure S4, Supplementary File 1). In addition, it could be shown that the erroneous density at the interface leads to a slight deviation of the molecular orientations ( Figure S5, Supplementary File 1). Note that the build-up of the density artefacts for realistic MTS setups evolves on a time scale much shorter than the duration of performed simulations ( Figure S6, Supplementary File 1).

The numerical artefacts stem likely from an increasing mismatch between the forces and the position of the atoms the forces are applied to. In line with this interpretation, the density artefacts were suppressed for a typical value of the outer time step *n _m_*∆ *t* = 10 fs when the mid-range forces were applied impulse-wise (TR RESPA scheme, blue line in Figure 2), i.e. the direction of the forces and the atom positions matched at the time point when the forces were applied. However, the TR RESPA scheme does not eliminate the resonance artefacts of non-stochastic thermostats, but only shifts them to longer outer time steps in this context ( Figure 3). Thus, the partitioning of the system into different time scales together with the choice of the time steps in the MTS integration remain delicate aspects of current MD implementations ^40^, also for impulse-wise MTS algorithms ^1, 2, 41^.

On the contrary, only small effects on the density profile could be observed for the SR scheme with pairlist update periods up to *n _p_*∆ *t* = 40 fs ( Figure 3 and Figure 4). Due to the large cutoff *R _l_* used here (i.e. 1.4 nm), the changes in the pairlist are small between updates and scale sub-linearly with *n _m_* ( Figure S3, Supplementary File 1). This observation is in line with the study of Krieger *et al.* ^42^, where they found a negligible energy drift when using a similarly low pair-list update frequency in a homogeneous system. When insisting on a non-stochastic thermostat, a rough performance analysis of the run time showed that for a realistic setup approximately half of the computational performance loss due to evaluating the mid-range forces at every time step could be partially compensated by a less frequent update of the pairlist ( Table 2).

**Table 2.** Comparison of run times for three simulation setups with different nonbonded force treatment: single-range scheme (SR) and twin-range scheme with constant-force application (TR CFA) using a Nosé-Hoover (NH) thermostat. All systems were simulated for 0.4 ns on 1 CPU with 4 cores. A time step of 2 fs was used. The pairlist was generated using the standard charge-group based algorithm of the GROMOS program. The simulated system consists of 2002 chloroform and 8129 water molecules.

Force splitting	*n _m_*	*n _p_*	Run time [h]
TR CFA	5	5	6.1
SR	1	5	11.4
SR	1	20	8.8

### Chemical composition and constant pressure

**Figure 5.**
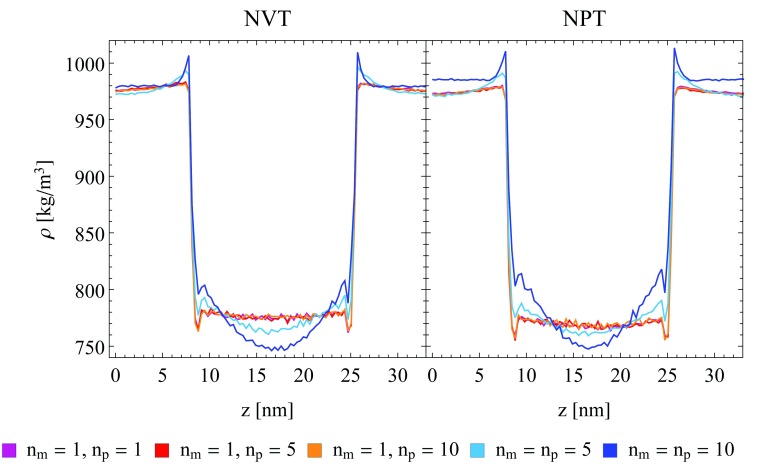
Density profile of the water-cyclohexane layer system under NVT conditions (left) or NPT conditions (right). A semi-aniostropic pressure coupling was applied in *x*/ *y*-direction using a Berendsen barostat, while the box length in *z*-direction remained fixed. A Nosé-Hoover (NH) thermostat was used in all setups. The single-range (SR) scheme (purple, red, and orange lines) and the twin-range scheme with constant-force application (TR CFA, light and dark blue lines) and three different update frequencies ( *n _p_* = 1, 5, 10) are compared. Running averages with a window of seven data points are shown.

In order to exclude that the observed artefacts are specific to the water-chloroform system, the densities at a water-cyclohexane interface in a layer system were investigated. The corresponding density difference plots with respect to a SR reference are given in Figure 4. They show a similar behaviour of the TR scheme compared to the one observed for the water-chloroform systems, with slightly less pronounced artefacts. In addition, imposing constant pressure (NPT) conditions in the simulation setup does not reduce the observed density artefacts ( Figure 5). The average pressure in the NVT simulations and the average volume in the NPT simulations are listed in Table S1 ( Supplementary File 1). Note that under NVT conditions, the observed density artefacts for the TR scheme lead to a reduction of the pressure as a function of the pairlist update frequency. As mentioned the box volume is slightly too small due to the chosen setup. Under NPT conditions and using the SR scheme, the box volume remains relatively constant. However, the semi-anisotropic pressure coupling in the *x*, *y*-direction with fixed *z*-dimension did not allow to preserve the system pressure at 1 atm for the elongated bilayer system. Separate scaling of the *z*-dimension did not improve the pressure control of the given system (data not shown). Using the TR scheme, the corresponding box volumes decrease as a function of the pairlist update frequency, in line with the NVT results.

### Geometry of the interface

In addition to planar interfaces, systems with a droplet of chloroform or cyclohexane in a water box were investigated. As can be seen in the right panels of Figure 4, density artefacts occur also at curved interfaces. Note that the corresponding plots contain more noise at small radii compared to the planar setups due to surface fluctuations, which influence the position of the centre of mass used as a reference. In addition, the surface fluctuations also give rise to a broadening of interface in terms of densities compared to the planar setup as can be seen in Figure S7 ( Supplementary File 1)

### Thermostatting method

**Figure 6.**
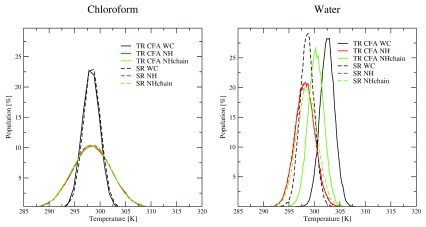
Histogram of the kinetic energy for chloroform (left) and water (right) using a weak coupling (WC, black), Nosé-Hoover (NH, red) and Nosé-Hoover chain (NHchain, green) thermostat (bin size 1 K). Note that a different number of molecules is simulated for chloroform (left) and water (right), which leads to a different width of the canonical distribution functions. For the twin-range (TR) scheme (solid lines), the mid-range forces were updated with the same period as the pairlist (i.e. *n _m_* ** = *n _p_* ** = 5). For the single-range (SR) scheme (dashed lines), the mid-range forces were evaluated every time step Δ *t* (i.e. *n _m_* ** = 1), whereas the pairlist was updated every 5th step (i.e. *n _p_* ** = 5).

The impact of the chosen non-stochastic thermostatting algorithm as well as the MTS integrator, and the energy distributions was investigated for the water-chloroform layer system. The WC thermostat does not give a canonical distribution of the kinetic energy by construction ^43^, whereas the NH and NHchain thermostats do. In Figure 6, the kinetic energy distributions within water and chloroform are shown for the different setups. For chloroform, the difference in the width of the distribution between WC and NH(chain) thermostats is clearly visible, whereas the average kinetic energy remains the same, despite the significant density gradient in this solvent. In water, on the other hand, a clear shift in the kinetic energy distribution towards a higher average value was observed for the TR CFA scheme compared to the SR scheme with the WC thermostat. This shift was less pronounced with the NHchain thermostat, and absent with the NH thermostat. These observations indicate that a temperature shift may occur due to MTS resonances. However, as the density increase occurred with all three thermostatting methods, but the temperature shift only with two of them, the latter cannot be the cause for the former. Therefore, one can conclude that the resonance artefacts caused by the TR scheme cannot be removed with thermostats that control the velocities globally. For this, one would have to control or perturb the velocities selectively as done in SD or as suggested in the literature by imposing isokinetic constraints or restraints on each degree of freedom ^21– 23^.

Zip containing simulation input files Click here for additional data file. Copyright: © 2019 Sidler D et al. 2019 Data associated with the article are available under the terms of the Creative Commons Zero "No rights reserved" data waiver (CC0 1.0 Public domain dedication).

## Conclusions

The effect of using the TR scheme with a constant force application (CFA) at solvent-solvent interfaces was investigated with layer and droplet systems of different composition (water-chloroform and water-cyclohexane). These systems represent simplified versions of a biological membrane and a protein in solution. The force splitting was found to lead to substantial MTS resonance artefacts at the interfaces for WC, NH and NHchain thermostats. It could be shown that the presence of the artificial density increase at the interfaces is independent of the interface geometry as well as the chemical composition of the system although the magnitude could vary. In addition, the diffusivity of the solvent molecules at the interface changed due to the density artefacts.

The resonance effects likely stem from a mismatch between the forces and the position of the atoms the forces are applied to. This hypothesis is supported by the fact that the pairlist changes only in the order of 1 % between updates and that the TR scheme with RESPA (where the forces are applied impulse-wise) does not eliminate the numerical artefacts but shifts them to longer time steps. The randomly applied stochastic forces of a SD thermostat seem to disrupt the MTS resonance build-up and thus successfully prevent the occurrence of density artefacts at the interfaces. Based on these findings, the use of TR CFA is not recommended for heterogeneous systems with WC, NH or NHchain thermostats. Instead, the SR scheme should be used, which did not show any significant density errors in the entire range of pairlist updates (analysed up to *n _p_*∆ *t* = 40 fs). Thus, the increase in computational cost for SR can be partially compensated by updating the pairlist less frequently (i.e. *n _p_*∆ *t >* 10 fs). Of course, there is a limit to the latter, as other errors are introduced by less frequent pairlist updates. By using only a single pairlist, the complexity of the implementation can be reduced, as the additional difficulties arising from efficient memory management of two (or more) pairlists can be avoided.

## Data availability

The data referenced by this article are under copyright with the following copyright statement: Copyright: © 2019 Sidler D et al.

Data associated with the article are available under the terms of the Creative Commons Zero "No rights reserved" data waiver (CC0 1.0 Public domain dedication). 

We are unable to provide the output files from the simulations directly due to file size. We instead provide the input files that when used as input with the GROMOS software package will generate the same results -

Dataset 1: Zip containing simulation input files 10.5256/f1000research.16715.d222949 ^44^

## Supplementary material

**Supplementary File 1: File containing the following supplementary figures and table:**

- **Figure S1**: Snapshots of different simulation setups.
- **Figure S2**: Phase separated density profile.
- **Figure S3**: Change in mid-range pairlist with respect to update frequency.
- **Figure S4**: Spatially resolved mean residence time.
- **Figure S5**: Molecular orientation at interface.
- **Figure S6**: Convergence of the density profiles.
- **Figure S7**: Droplet density profiles.
- **Table S1**: Average box volume *V* and pressure *P* for the water-cyclohexane layer system with corresponding standard deviation Δ *V* and Δ *P*.
