# Supplementary material for: Density artefacts at interfaces caused by multiple time-step effects in molecular dynamics simulations
Source: F1000Res. 2019 Mar 8;7:1745. Originally published 2018 Nov 5. [Version 3] doi: 10.12688/f1000research.16715.3 (PMC6441880; doi:10.12688/f1000research.16715.3)
Supplement: Supplementary file 2 [file f1000research-7-20224-s0001.tgz › 4ec2748c-b8dd-4d92-8be3-309ab509613d_Supplementary_File_1_v3.pdf]

Supplementary Material: Density Artefacts at Interfaces Caused by Multiple Time-Step Effects in Molecular Dynamics Simulations

Dominik Sidler, Marc Lehner, Simon Frasch, Michael Cristòfol-Clough and Sereina Riniker*

*Laboratory of Physical Chemistry, ETH Zürich, Vladimir-Prelog-Weg 2, 8093 Zürich, Switzerland, sriniker@ethz.ch, www.riniker.ethz.ch

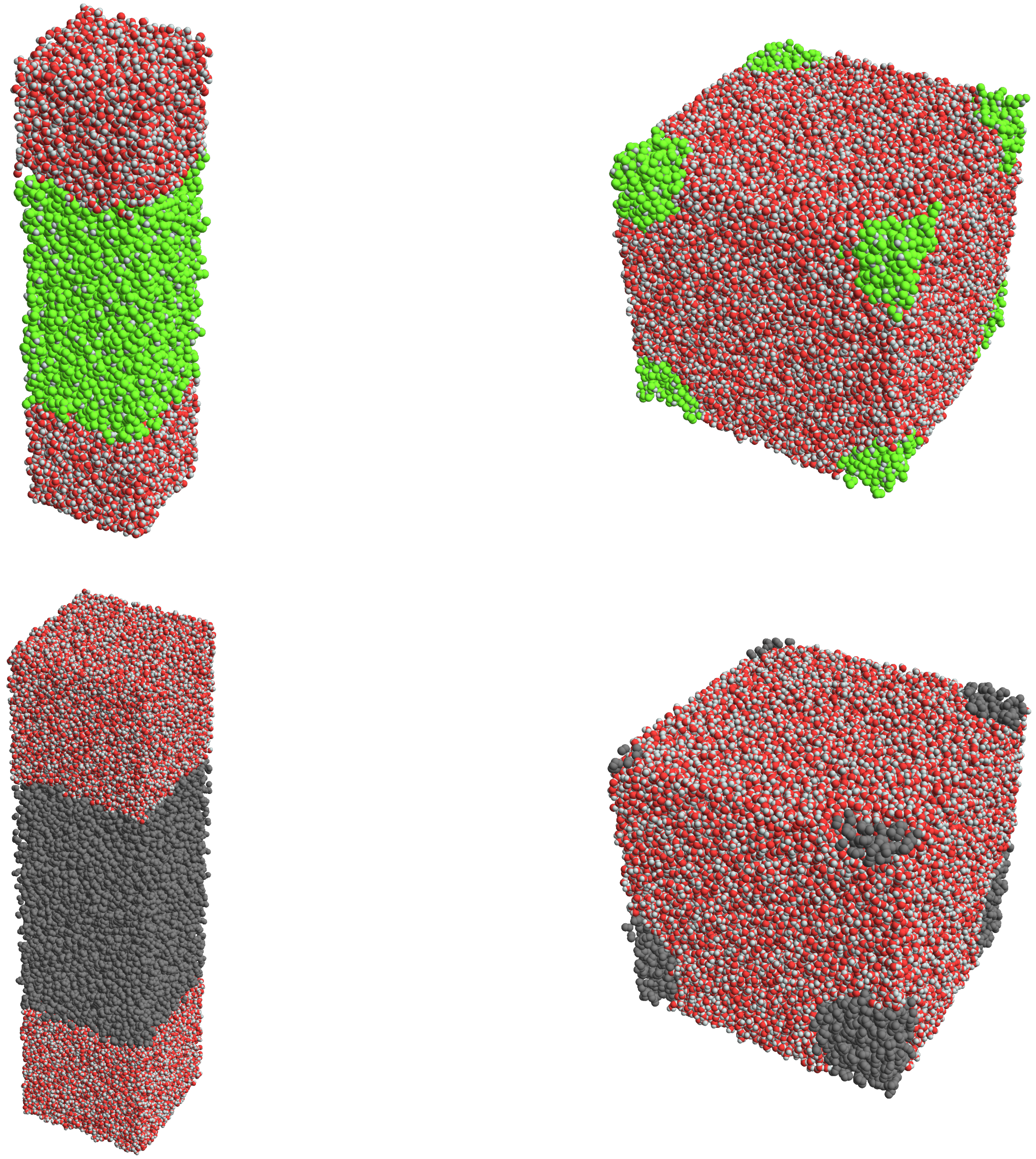

Figure S1: Snapshots of the layer (left) and droplet (right) system setups with water-chloroform (top) and water-cyclohexane (bottom).

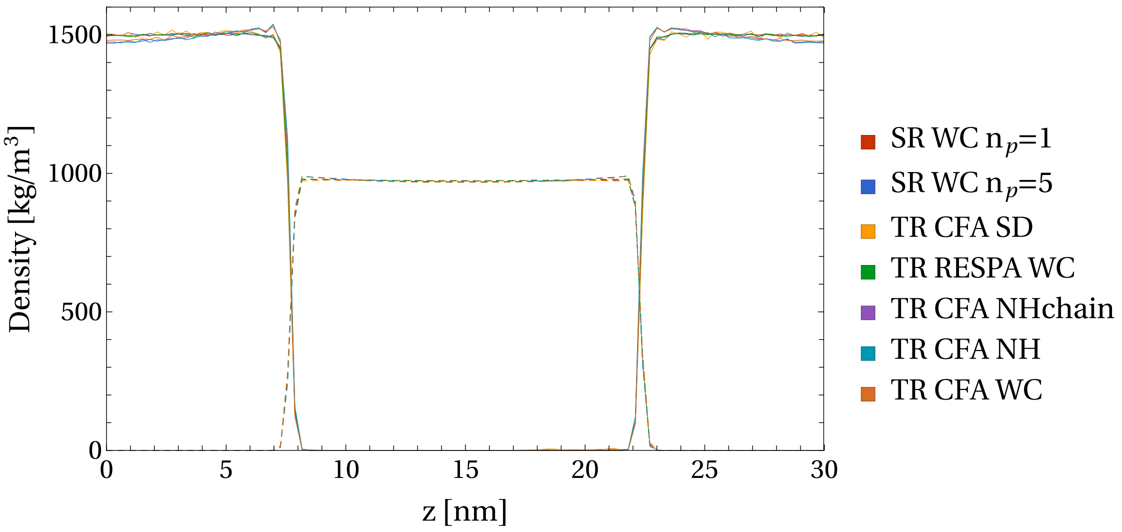

Figure S2: Density profile for the water-chloroform bilayer system, where each phase is treated individually. All TR simulations were performed using $n_p = 5$. Solid lines correspond to chloroform and dashed lines correspond to water. Note that no mixing of the phases occurs and the interface is located at a relatively narrow z-range, which indicates that the shape and position of the interface evolves little over the chosen simulation time.

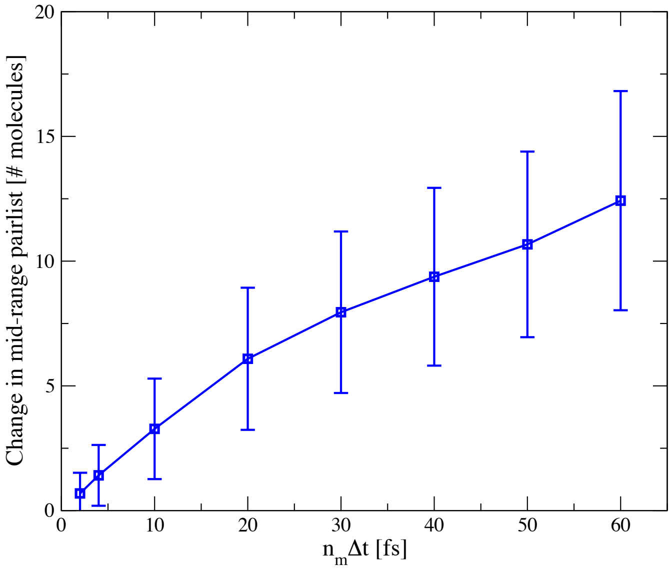

Figure S3: Average change (in number of molecules) of the mid-range pairlist between two pairlist updates (i.e. every n_m steps). A cubic SPC water box of 1000 molecules was simulated under NVT conditions using the TR CFA algorithm with a Nosé-Hoover thermostat. The mid-range shell was bound by $R_s = 0.8$ nm and $R_l = 1.4$ nm. After 0.4 ns equilibration time, all molecule coordinates were written out every outer time step $n_m\Delta t = n_p\Delta t$ during 1000 consecutive pairlist updates. One random molecule was selected and the difference between two consecutive pairlist updates was tracked for the mid-range pairlist. The average number of water molecules in the mid-range pairlist is 305.

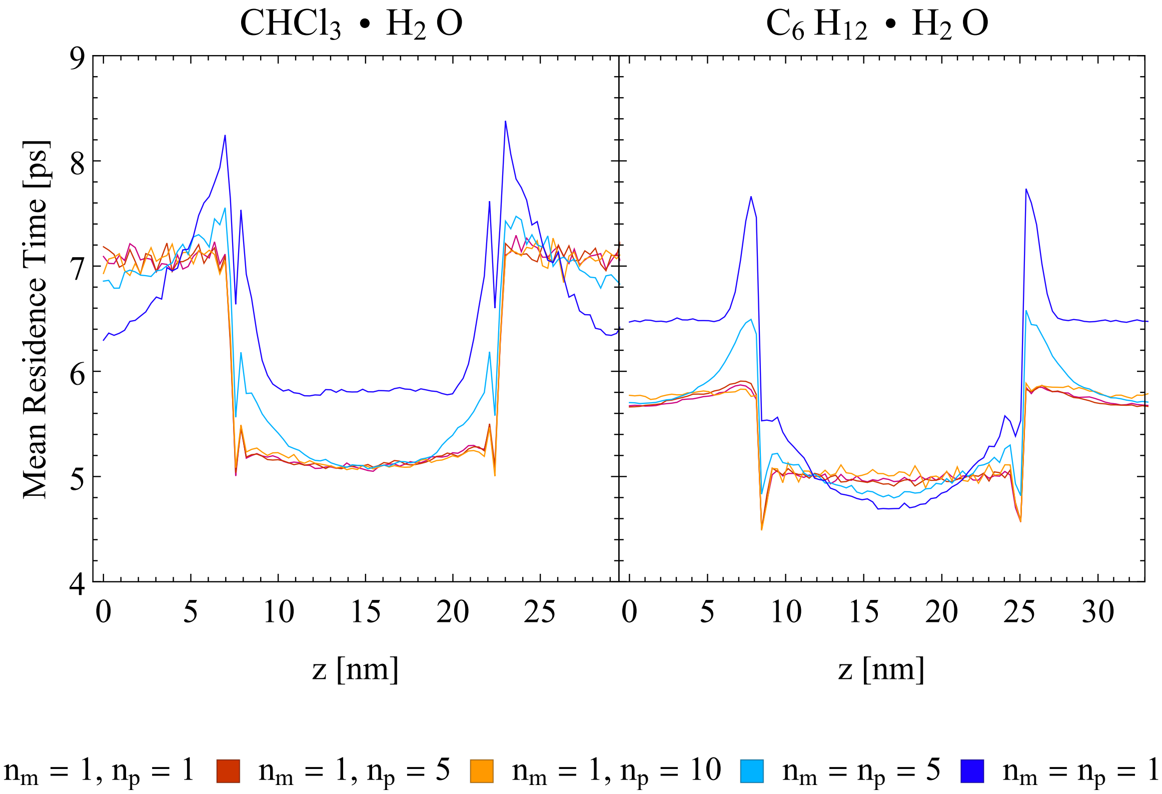

Figure S4: Mean residence time as a function of the z-coordinate for the two investigated layer systems with water-chloroform (left) and water-cyclohexane (right) using a Nosé-Hoover thermostat. 100 vertical slices of 0.3 nm width were used. Simulations were performed under NVT conditions over 1 ns with the SR scheme (purple, red and orange lines) and the TR CFA scheme (light and dark blue lines) with three different update frequencies ($n_p = 1, 5, 10$).

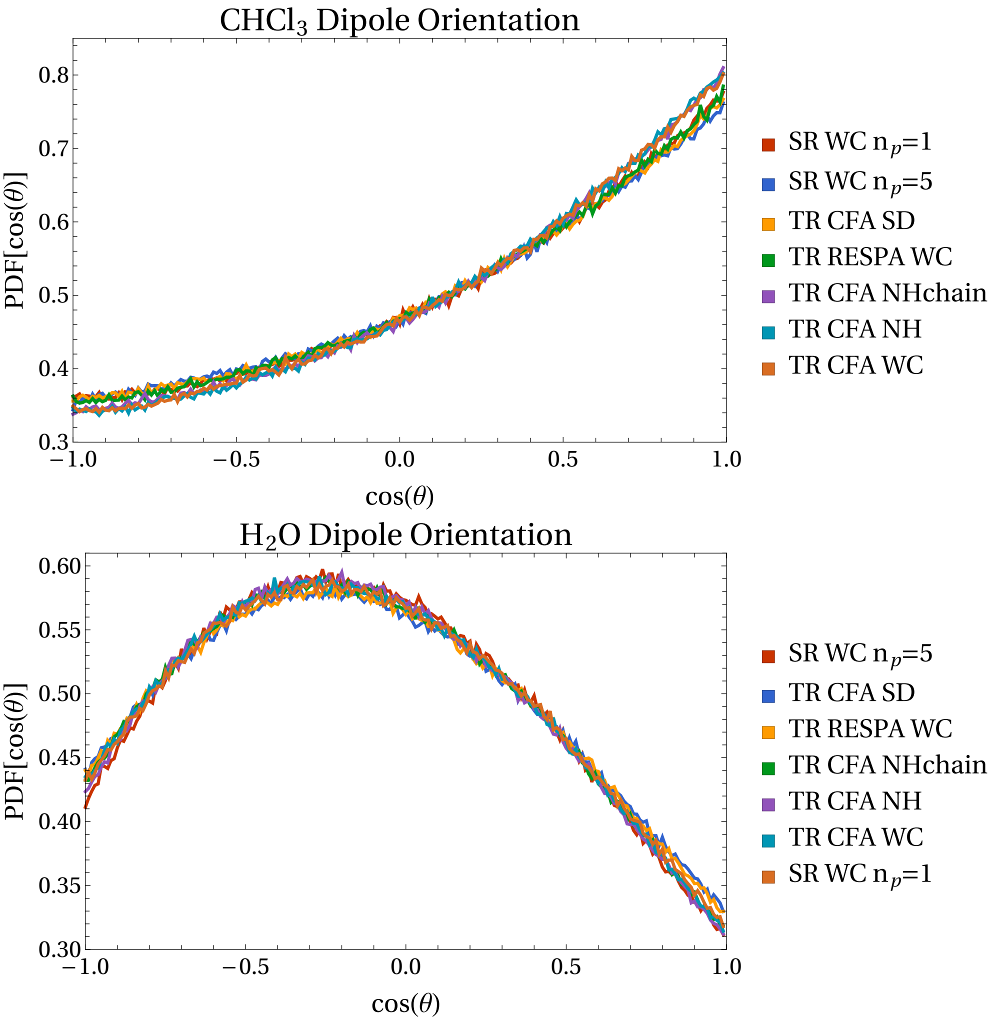

Figure S5: Probability density function (PDF) of the angular distribution for the chloroform (top) and water (bottom) molecules with respect to the interface normal vector $\vec{n}_j$ (along z-axis). For the water and chloroform molecules j the dipole moment $\vec{d}_j$ was used for the calculation of $\cos(\theta_j) = \frac{\vec{n}_j \cdot \vec{d}_j}{n_j d_j}$. For the calculation of the molecular orientations, a slice of 2 nm thickness was considered at the interface for each phase. Note that the density artefacts of the TR setups are accompanied with a small change of the angular distribution in chloroform.

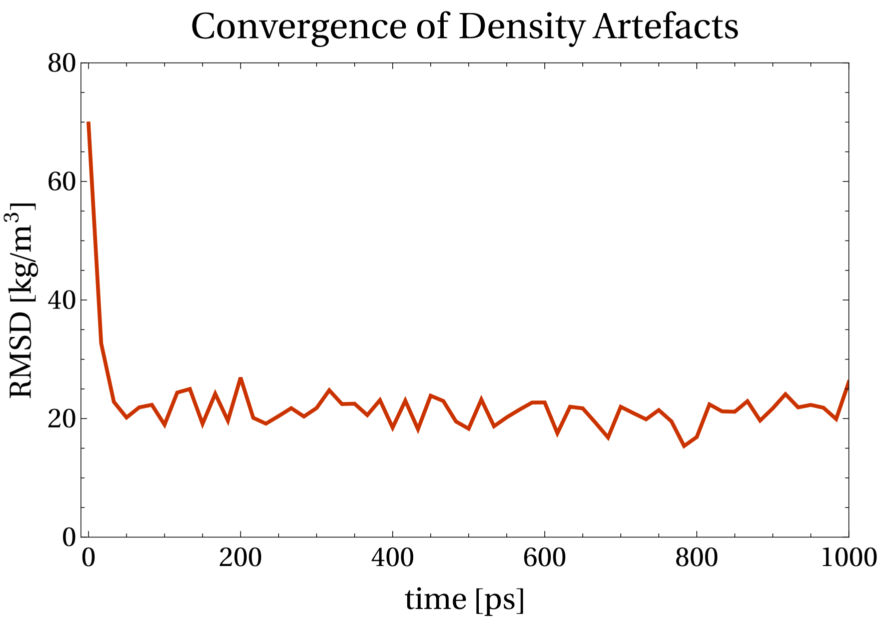

Figure S6: The root-mean-square deviation ($RMSD_t$) of the chloroform density ρ was calculated for each time-step t with respect to the average density $\bar{\rho}$ of all frames as given in Eq. (1) below. The displayed result corresponds to the water-chloroform bilayer simulated with the TR CFA algorithm using $n_m = n_p = 5$ and a Nosé-Hoover thermostat. This graph indicates that the density artefacts (deviations) occur on a much shorter time scale than the simulation time of the systems (1 ns). Therefore, the simulation results are considered to be converged.

$$\mathrm{RMSD_t} = \sqrt{\sum_{i=1}^{b} (\bar{\rho}_i - \rho_{it})^2} \tag{1}$$

t : simulation timestep

b : number of bins along z ($b = 200$)

i : bin number

$$\rho_{it} = \frac{1}{V_i} \sum_{k=1}^{N_i} m_k$$

$$\bar{\rho}_i = \frac{1}{T} \sum_{t=0}^{T} \rho_{it}$$

T : total number of timesteps considered

N_i : number of molecules within bin i

V_i : volume of bin i ($V_i := 12.125$ [nm^3])

m_k : mass of molecule k

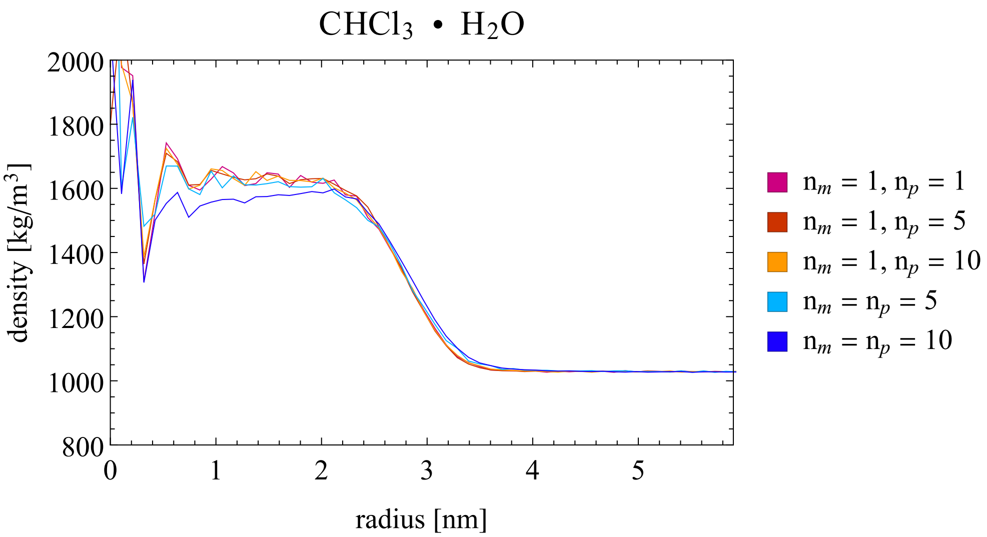

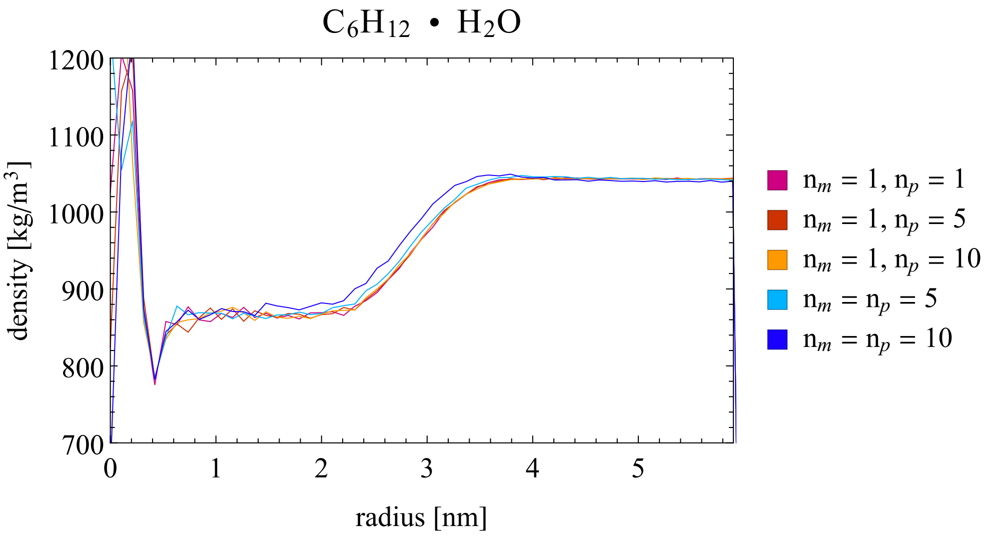

Figure S7: The density profile with respect to droplet radius is shown for the chloroform in water (top) and for the cyclohexane in water droplet (bottom) using a Nosé-Hoover thermostat under NVT conditions. Note that the transition region between the two phases is broadened, in comparison with the planar systems. This broadening is caused by deviations of the droplet from its ideal spherical symmetry during runtime.

Table S1: Average box volume V and pressure P for the water-cyclohexane layer system with corresponding standard deviation ΔV and ΔP. The first 200 ps were discarded for equilibration. Averaging was performed over production runs of 1 ns length.

Condition	n_m	n_p	$V \pm \Delta V$ [nm^3]	$P \pm \Delta P$ [atm]
NVT	1	1	2425	85.2 ± 34.0
	1	5	2425	87.7 ± 34.8
	1	10	2425	81.7 ± 34.7
	5	5	2425	46.6 ± 33.0
	10	10	2425	-31.1 ± 35.6
NPT	1	1	2451 ± 1.6	10.9 ± 29.0
	1	5	2440 ± 1.8	9.3 ± 29.8
	1	10	2439 ± 1.5	9.6 ± 30.0
	5	5	2432 ± 1.6	11.7 ± 29.4
	10	10	2410 ± 1.7	36.3 ± 30.4
