# Supplementary material for: Density artefacts at interfaces caused by multiple time-step effects in molecular dynamics simulations
Source: F1000Res. 2019 Mar 8;7:1745. Originally published 2018 Nov 5. [Version 3] doi: 10.12688/f1000research.16715.3 (PMC6441880; doi:10.12688/f1000research.16715.3)
Supplement: Peer review history

# Peer review: Reviewer response for version 3

**DOI:** 10.5256/f1000research.20224.r45443

**Published:** 2019-03-22

**Authors:** Bojan Zagrovic, Daniel Braun

**Affiliations:** 1 Department of Structural and Computational Biology, Max F. Perutz Laboratories, University of Vienna, Vienna, Austria

The authors have adequately addressed our comments.

We have read this submission. We believe that we have an appropriate level of expertise to confirm that it is of an acceptable scientific standard.

# Peer review: Reviewer response for version 2

**DOI:** 10.5256/f1000research.19532.r43226

**Published:** 2019-01-29

**Authors:** Bojan Zagrovic, Daniel Braun

**Affiliations:** 1 Department of Structural and Computational Biology, Max F. Perutz Laboratories, University of Vienna, Vienna, Austria

The authors have adequately addressed most of the concerns raised in the original review. However, there are still some open questions that need to be clarified before the manuscript is indexed. The numbers below refer to the numbering in the original review.

 Major comments: 

 Comment 1. It is not fully clear what the chloroform density in Figure S6 is and how it is calculated. Assuming chloroform density describes the density of the chloroform component, how was the respective volume calculated? Does the plot really show data for each frame, as stated, or is the data averaged in some way? Secondly, our original concern related to the convergence of the phenomena at hand, i.e. the buildup of the density artefacts. In the main text, it is stated that a density increase at the interface "was accompanied by a density decrease further away from the interface due to the constant volume conditions" and not by a change in density of the whole component (as also shown in Figure S2). Therefore, it is not clear how the quantity given in Figure S6 relates to density artefacts.

 Comment 2. Regarding the box size in constant volume simulations, we believe that there is an issue that needs to be analyzed in more detail. In general, it is common practice to use constant pressure simulations to determine the appropriate box size for constant volume simulations after finalizing the MD setup, including the definition of integrator, thermostat, pairlist specifications, etc. We acknowledge that in a study which aims at comparing different MD setups, the researcher faces a dilemma. On the one hand, equilibrating the box size for each MD setup entails a variation in the overall density. On the other hand, comparing all MD setups at the same box size entails a variation in pressure, with a potentially even more severe influence on a number of properties, e.g. system dynamics and, as relevant here, also on the extent of the observed artefacts in the present study. The authors should address this topic in more detail. In the Methods section, this should include a detailed explanation of the problem at hand and the choices that the authors have made (variation in pressure vs. variation in density). In particular, a list of the overall values of density/pressure for each system should be included. Furthermore, the authors should provide an estimate of the influence of their choice of how to set up the NVT simulations on system properties and foremost on the density artefacts in their paper.

 Comment 4. Figure 2 still shows somewhat systematically conserved fluctuations (i.e. present across different simulation systems). This was even more obvious in the original figure. Independent of bin size, where do these (apparently) conserved fluctuations come from?

 Minor comments:

 Comment 5. The downsides of SR increased pairlist update intervals should be discussed.

We have read this submission. We believe that we have an appropriate level of expertise to confirm that it is of an acceptable scientific standard, however we have significant reservations, as outlined above.

# Response

**Published:** 2019-03-01

**Authors:** Sereina Riniker

**Affiliations:** ETH Zürich, Switzerland

Major comments:

(1) We have adapted the caption of Figure S6 and added Equation (1), which was used in the calculation, in the Supplementary Material.

(2) As the density artefacts were observed in both NVT and NPT simulations, we can rule out this factor as a cause for the occurrence of the artefacts. Nevertheless, we have added a sentence in the Methods section describing the issue and our rationale for choosing the equilibration procedure. In addition, we have added Table S1 in the Supplementary Material with the pressure and box volume in the NVT and NPT simulations of the water-cyclohexane system. The data is discussed in the Section "Chemical composition and constant pressure".

(4) The apparent fluctuations are due to a combination of the relative orientation of the molecules at the interface (see Figure S5), the granularity of the atoms in a classical force field, and the binning.

Minor comment:

(5) We have added a short discussion of this issue in the Conclusions.

# Peer review: Reviewer response for version 1

**DOI:** 10.5256/f1000research.18272.r40235

**Published:** 2018-12-03

**Authors:** Nico. F. A. van der Vegt

**Affiliations:** 1 Eduard-Zintl-Institute of Inorganic and Physical Chemistry, TU Darmstadt, Darmstadt, Germany

This is an interesting and well-conducted study on density artefacts observed in molecular dynamics simulations that use multiple time-step integration algorithms to study liquid-liquid interfacial systems. The authors nicely show how depending on choices made for pairlist updates and thermostatting algorithms twin-range schemes can lead to severe density artefacts at (water-chloroform and water-cyclohexane) liquid-liquid interfaces. It is shown that these density artefacts cannot be remedied when widely used thermostats (WC, NH, NH-chain) are used that control the velocities globally. Do the density artefacts observed in these systems disappear when a standard, stochastic thermostat is used instead?

I have read this submission. I believe that I have an appropriate level of expertise to confirm that it is of an acceptable scientific standard.

# Response

**Published:** 2019-01-10

**Authors:** Sereina Riniker

**Affiliations:** ETH Zürich, Switzerland

We thank the reviewer for this suggestion. We have performed additional simulations with a stochastic thermostat, which show that the artifacts disappear with this thermostat (see updated Figures 2 and 3). We have adapted the figures, text and conclusions accordingly.

# Peer review: Reviewer response for version 1

**DOI:** 10.5256/f1000research.18272.r40231

**Published:** 2018-11-23

**Authors:** Bojan Zagrovic, Daniel Braun

**Affiliations:** 1 Department of Structural and Computational Biology, Max F. Perutz Laboratories, University of Vienna, Vienna, Austria

In the article "Density artefacts at interfaces caused by multiple time-step effects in molecular dynamics simulations" by Sidler et al., the authors investigate the possible artefacts introduced by multiple time-step (MTS) integration methods for molecular dynamics (MD) simulations with respect to the local density and diffusivity at simulated interfaces between polar and apolar media. The simulated systems include chloroform-water and cyclohexane-water mixtures, both arranged in either planar-layer or droplet conformations. For all simulated systems, the authors report artificial density increases at the interface of the two liquids, whereby the degree of the observed effect depends on the MTS algorithm used. The density artefacts are studied mainly in the NVT ensemble, but are shown to also appear in the NPT ensemble. Moreover, some of the investigated thermostats introduce a shift in the distribution of kinetic energies in the water component. Based on these findings, the authors recommend the usage of the single-range treatment of the non-bonded interactions with lower pairlist update frequencies, which are less sensitive with respect to the discussed artefacts as compared to the widely used twin-range schemes. The study makes a valuable contribution towards the improvement of the existing MD methods when it comes to both accuracy and computational performance. The observed artefacts are certainly pronounced and cannot be ignored. On the other hand, certain aspects of the study lack the necessary explanations or depth of exploration and thus have to be improved accordingly.

 Major comments

  

1. The length of individual simulations is relatively short by state-of-the-art standards. This, of course, is not a major issue if the phenomena at hand converge comparatively quickly, as indeed implied by the authors. However, for completeness, the convergence should be quantitatively demonstrated.
2. For all NVT simulations, the authors should perform preliminary runs of several 100 ps in the NPT ensemble for density equilibration, as well as several 100 ps of equilibration in the studied ensemble before the production runs. The overall density in the present NVT simulations is determined by the authors in the course of the setup of the simulation boxes. It is conceivable that the ideal simulated density would vary with respect to the integration method, time-step or thermostating method. As a consequence, some simulated NVT systems could exhibit too-low or too-high overall density, which on its own could introduce or at least influence some of the observed artefacts.
3. The microscopic origin(s) of the density artefacts should be investigated in greater detail. In particular, an in-depth investigation of the molecular structure at the interfaces would be instructive. For example, are the relative orientations of the chloroform/cyclohexane molecules in the areas of higher density different from those in the bulk? How about water? If so, how can this be related to the algorithmic differences? This analysis should, *inter alia*, involve a decomposition of density profiles into chloroform (or cyclohexane) and water components, but also more elaborate structural analyses (for an example of such analysis, please refer to ^1^). 
4. The density profiles in Figure 2 exhibit curious short-range fluctuation patterns, which even appear somewhat periodic. The authors should analyze the origin of these patterns and potentially link them with the main analysis at hand.
5. The authors should analyze and comment more extensively on the asymmetric appearance of the TR-CFA-WC and TR-CFA-NH density profiles in Figure 3 with respect to the planar layers at high values of the pairlist update period. They comment that the asymmetry may be due to insufficient sampling, but given how extensive and relevant for the central argument of the paper it may be, it should be analyzed in more detail. In an ideal case (i.e. if largely symmetric) the density profiles could be averaged over the two sides for greater statistical power.

Minor comments

1. The CFA and RESPA approaches need to be explained in greater detail in the introduction. This seems especially important since RESPA seems to be much less prone to density artefacts than CFA.
2. The colors are referenced wrong in the caption of Figure 4.
3. The caption of Figure 3 contains an "n" without an index.
4. The authors should explicitly describe how the density was calculated even for planar systems (bin size, averaging volume etc.).
5. While the advantages of updating the Pairlist less frequently are clearly illustrated, in the conclusions section the authors should also discuss in more detail the potential downsides of such a procedure.

We have read this submission. We believe that we have an appropriate level of expertise to confirm that it is of an acceptable scientific standard, however we have significant reservations, as outlined above.

## References

[1] HantalGSegaMKantorovichSSchröderCJorgeM: Intrinsic Structure of the Interface of Partially Miscible Fluids: An Application to Ionic Liquids. *The Journal of Physical Chemistry C*.2015;119(51) : 10.1021/acs.jpcc.5b09810 28448-28461 10.1021/acs.jpcc.5b09810

# Response

**Published:** 2019-01-10

**Authors:** Sereina Riniker

**Affiliations:** ETH Zürich, Switzerland

Major comments:

1. We have checked the convergence, which is reached within 100 ps (new Figure S6 in the Supporting Information), well below the simulation time. 
2. The individual solvent boxes were equilibrated in the NPT ensemble, before combining them in the layer system. In the absence of artefacts (e.g. with a single-range scheme), the density profiles remain perfectly constant in the NVT ensemble. We consider this to be a more suited starting configuration than the layer system equilibrated additionally in the NPT ensemble with the twin-range scheme, because it shows the full extent of the artefacts introduced by the latter.
3. We thank the reviewers for this suggestion. We have investigated the orientation of the solvent molecules at the interface, which is affected by the artefacts from the MTS algorithm. The results are presented in the new Figure S5 in the Supporting Information and discussed in the text. A decomposition of the density profiles is shown in the new Figure S2.
4. These "fluctuations" stem from the binning of the density profiles. We have changed Figure 2 to have a consistent bin size for all curves.
5. These fluctuations arise mainly due to the finite box size. We have adapted the text accordingly.

Minor comments:

1. We have added additional explanations of the MTS algorithms with equations.
2. We thank the reviewer for spotting this. We have corrected the caption of Figure 4 accordingly.
3. We thank the reviewer for spotting this. We have corrected the caption of Figure 3 accordingly.
4. We have added these details in the Methods section.
5. Due to the new results with the stochastic dynamics thermostat, the conclusions have been adapted.
